# Influence of Processing and Stabilizer Selection on Microstructure, Stability and Rheology of Emulsion-Based Semisolid Formulations

**DOI:** 10.3390/pharmaceutics17091221

**Published:** 2025-09-20

**Authors:** Ruochen Yang, Xin Yi Tee, Sendhil Kumar Poornachary, Elena Simone, Pui Shan Chow

**Affiliations:** 1 Institute of Sustainability for Chemicals, Energy and Environment, Agency for Science, Technology and Research (A*STAR), 1 Pesek Road, Jurong Island, Singapore 627833, Singapore; yang_ruochen@isce2.a-star.edu.sg (R.Y.); tee_xin_yi@isce2.a-star.edu.sg (X.Y.T.);; 2 Department of Applied Science and Technology, Politecnico di Torino, 10129 Torino, Italy; elena.simone@polito.it

**Keywords:** emulsion, topical formulation, MDG, stabilizer, microstructure, rheology, physical stability, manufacturing process

## Abstract

**Background/Objectives**: Emulsion-based semisolid formulations are important delivery systems for many applications, including pharmaceuticals, cosmetics and food. The manufacturing process for such formulations typically involves a series of heating, cooling, mixing and emulsification steps. Stabilizing agents are usually included in such formulations, as emulsions are intrinsically unstable and are prone to various destabilization mechanisms. Precise control of each processing parameter and the selection of an appropriate stabilizing agent are essential for delivering products with long-term stability and the desired properties. In this study, the effects of emulsification temperature and the selection of the stabilizing agent on key product attributes were investigated to enable improved design and optimization of both the formulation and manufacturing process. **Methods**: Model emulsion systems containing propylene glycol (PG) as the dispersed phase and mineral oil as the continuous phase were prepared at different emulsification temperatures to cover both pre-crystallization and post-crystallization regimes. Three stabilizing agents, namely mono-and-diglyceride (MDG), neat monoglyceride (MG) and neat diglyceride (DG), were studied. Their crystallization behavior was first examined to determine crystallization temperatures and crystal morphologies. The resulting emulsion samples were then characterized in terms of their microstructure, physical stability and rheological properties. **Results**: The emulsions prepared under post-crystallization conditions exhibited better physical stability, higher rheological parameters (crossover stress and viscosity) and a more rigid microstructure compared to those formed under pre-crystallization conditions, regardless of the stabilizer used. Rheological properties were found to corelate well with physical stability. In the pre-crystallization regime, poor stability could partially be mitigated by lowering the emulsification temperature. MG was generally more effective than DG in stabilizing the emulsions and led to higher rheological properties, despite both crystallizing into the same polymorph within the system. This difference in performance was attributed to variations in the crystal morphology and spatial distribution within the emulsion. Notably, the MG-stabilized emulsions also displayed a self-hardening effect during storage. **Conclusions**: The selection of the appropriate stabilizing agents and processing conditions tailored to the specific system is critical for the successful manufacture of emulsion-based semisolid products with an optimized performance.

## 1. Introduction

Semisolid dosage forms such as ointment, creams and gels are used for the topical administration of active pharmaceutical ingredients (APIs), and such delivery systems offer various advantages, including ease of application, better patient compliance, targeted delivery, long residence times and reduced systemic toxicity [1,2,3,4]. Many of these semisolid dosage forms are either water-in-oil (w/o) or oil-in-water (o/w) emulsions, which offer the flexibility of delivering APIs with different hydrophobicity. Simple emulsions contain two thermodynamically immiscible liquid phases (the oil phase and the aqueous phase), with one phase being the continuous or the external phase and the other the dispersed or internal phase [5]. Emulsion-based semisolid dosage forms are more complex systems that contain many components. The microstructure of these systems is often complex with multiple phases. Given that emulsion systems are intrinsically unstable, stabilizing agents such as surfactants or solid crystals are often added to semisolid formulations to kinetically stabilize the dispersed droplets and allow other key product attributes to be tuned at the same time [6,7,8,9]. There are two widely recognized mechanisms for kinetic stabilization: network stabilization, where stabilizing agents form a viscoelastic network throughout the continuous phase [10,11], and Pickering stabilization, where interfacially adsorbed solid particles reduce droplet coalescence [10]. Several studies have argued that Pickering stabilization is more effective than network stabilization [12,13], although they can occur in the same system at the same time [13].

Many studies have shown that microstructure of topical formulations can influence various essential physical properties, including rheology, physical stability and sensory properties [14,15,16,17,18]. This study will focus on how processing parameters, specifically emulsification temperature and the choice of stabilizer, can affect important physical properties of emulsion-based topical formulations. Other than these physical properties, microstructure has also been shown to influence active release, usually measured from in vitro release tests (IVRTs) of topical formulations. Active release is a key product attribute for semisolid dosage forms, as it is necessary to demonstrate Q3 (microstructure) similarity [19]. Factors that can affect IVRTs include viscosity, polymorphism, API particle sizes when dispersed as particles and the size of the dispersed phase in the emulsions, to name a few [20,21], and both the formulation and processing conditions have been shown to affect the structure. Wan et al. reported that by formulating pseudolaric acid B (PAB) as a microemulsion, the amount of API retained on the skin and its permeation rate both increased compared to those for the gel formulation. This was due to the reduced size of the dispersed phase [22]. Xu et al. reported that the drug release from an ointment formulation of acyclovir followed a logarithmic kinetic model and the release constant in the model was dependent on the drug loading, API particle size and composition of mineral oil in the ointment base [20]. Yuan et al. showed that addition of hyaluronic acid as a permeation enhancer reduced in vitro drug release, although skin permeation and retention were both increased [23]. Raghavan et al. reported that for emulsions, the drug release rate varied depending on the sequence of oil phase addition and aqueous phase addition [21]. Chow et al. reported that a higher amount of lidocaine was released from an emulsion cream when additional shear was applied at 35 °C compared to that for samples where additional shear was applied at 25 °C [24]. The rationale was that more severe disruption of the microstructure would occur when shear was applied at a lower temperature, as the emulsifying wax will already have crystalized and encapsulated the active phase [24]. However, in another study on lidocaine ointment, it was reported that despite the differences in bulk viscosity and crystallite (encapsulated by MDG) sizes, the drug release for samples prepared with different processing parameters was similar [25]. This was suggested to be due to the direct diffusion of the API from free droplets into the receptor compartment in the in vitro release test setup [25]. Dong et al. reported am inverse correlation between the cumulative amount of drug release and viscosity for an emulgel formulation of terpinen-4-ol [26]. Wong et al. formulated poly (ethylene oxide) (PEO)-based hydrogels using different methods of drug loading, which affected the drug release profiles. Immediate release occurred when the API was incorporated into microporous spaces, and prolonged release occurred when the API was embedded into the polymer matrix [27]. Fanse et al. studied the drug release from two commercial levonorgestrel intrauterine systems (LNG-IUSs) and an in-house LNG-IUS. Differences in drug release were observed, and the release kinetics correlated well with their microstructures, characterized using advanced imaging techniques [28]. In another study on LNG-IUSs, higher polymer crosslinking density and lower crystallinity were correlated with faster drug release [29].

Mono-and-diglyceride (MDG) is a commonly used stabilizer in many pharmaceutical and cosmetic topical formulations and other food products [30,31]. Commercially available MDG is a mixture of mono, di and triglycerides (trace amounts) produced through esterification of glycerol and fatty acids. The fatty acids used are often a mixture of stearic acid and palmitic acid. As per the United States Pharmacopeia-National Formulary (USP-NF), MDG should contain no less than a 40% monoglyceride content. Furthermore, based on the ester bond position, monoglycerides (MGs) and diglycerides (DGs) can exhibit positional isomers such as 1-MG, 2-MG, 1,2-DG and 1,3-DG [32]. The crystallization behavior of neat glycerides and that in oleogels have been widely studied [33,34,35,36,37,38,39]. Different polymorphs are reported for neat glycerides [40]. Upon cooling, the first liquid-to-solid transition occurs at a temperature named the Krafft temperature when the isotopic fluid is converted into an inverted lamellar phase ($L_{\alpha }$), where the glycerol head groups are packed in a hexagonal way and the acyl chains remain largely amorphous. Upon further cooling to below the Krafft temperature, the inverted lamellar phase is converted into the sub-α phase (also referred to as the β’ phase or α gel) with orthorhombic subcells [33]. Both phases are unstable and can transform into the stable β crystalline form with a triclinic subcell type over time. Destabilization of MG-stabilized emulsions is often associated with a transition from the metastable phases, including both the inverted lamellar and sub-α phases, to the stable β crystalline phase [33]. Controlling the polymorphic form of the stabilizing agent in emulsion formulations is essential, as it can affect important product attributes such as textural properties [41], appearance [41] and physical stability [42]. 

Despite various studies on the crystallization of neat glycerides [33,34,35,36,37,38,39], there are limited reports on the crystallization behavior or crystal structure of MDG as a mixture. Kushwah et al. [32] studied the effects of aging and lot-to-lot variability on the crystalline structure of MDG. It was found that different batches contained different percentages of the sub-α and β phases, and the percentage of the β phase tended to increase with aging. Ali et al. [12] studied the crystallization of commercial MDG from paraffin oil and reported that the MG component mainly crystalized into the $L_{\alpha }$ phase, with a plate-like morphology, whereas DG directly crystallized into the stable β crystalline form, with a spherulite morphology. In a polyethylene glycol 400 (PEG 400)/paraffin oil emulsion, MG crystals mainly adsorbed onto the PEG droplet surface, whereas DG crystals resided in the bulk oil phase. Other studies have focused on the effects of the manufacturing process and formulation parameters on the product attributes for semisolid formulations that contain MDG [25,43,44,45]. Chow et al. [25] reported that the long-term stability of a model ointment stabilized with MDG could be enhanced by adding the dispersed phase into the continuous phase at a lower temperature. It was hypothesized that a lower addition temperature led to better encapsulation of the dispersed phase by the MDG crystals. Saremnejad et al. [44] developed an aerated nonaqueous foam by using MDG as the surfactant and achieved the desired formulation attributes with an MDG content of 10 wt%. It was reported that foamability and foam stability increased with increasing MDG content. Wang et al. reported that MDG promoted the crystallization of anhydrous milk fat and increased the solid fat content of aerated emulsions [45]. However, the existing literature has mainly focused either on the crystallization behavior of MDG from melts or solutions [12,32], without investigating its role as a stabilizer in emulsions, or on the macroscopic properties of MDG-stabilized emulsions, without addressing the underlying mechanism of emulsion stabilization by MDG [25,43,44,45]. In addition, the individual contributions of MG and DG, specifically how each component crystallizes and stabilizes the emulsions under different processing conditions, have not been thoroughly studied. Here, the effects of processing and formulation factors on macroscopic product attributes were studied in MDG-containing emulsions, with a focus on the microscopic behavior of MDG, and its stabilizing mechanism was also proposed.

In this study, the model system used was an emulsion system consisting of propylene glycol (PG) droplets dispersed in mineral oil, with MDG as the stabilizing agent. Crystallization of MDG from mineral oil was investigated to determine the crystallization temperature of the individual components, namely MG and DG, and their crystal morphologies and polymorphic forms. Emulsions were then prepared with different T_e_ values selected based on the crystallization temperatures that were determined previously. The effect of T_e_ on the resultant emulsion microstructure was analyzed using optical microscopy and correlated with the formulated product attributes, including rheology and stability. Finally, the effects of T_e_ on the stabilization efficiency of neat MG and DG were investigated separately to determine their individual contribution towards the emulsion stability. The findings reported here will help formulation scientists to design emulsion-based topical formulations better with optimized formulations and manufacturing processes tailored to different systems.

## 2. Materials and Methods

### 2.1. Materials

Light mineral oil (NF/FCC, CAS No. 0842-47-5) and propylene glycol (CAS No. 57-55-6) (Ph. Eur-grade) (PG) were purchased from Thermo Fisher Scientific (Loughborough, UK). Mono-and-diglyceride (Geleol™) (CAS No. 85251-77-0) (MDG) was purchased from Gattefosse (Saint Priest, France). Distilled monoglyceride (CAS No. 122-94-4) (MG) was supplied by Chemsino Industry Co., Ltd. (Zhengzhou, China).

### 2.2. Emulsion Preparation

**Figure 1:**
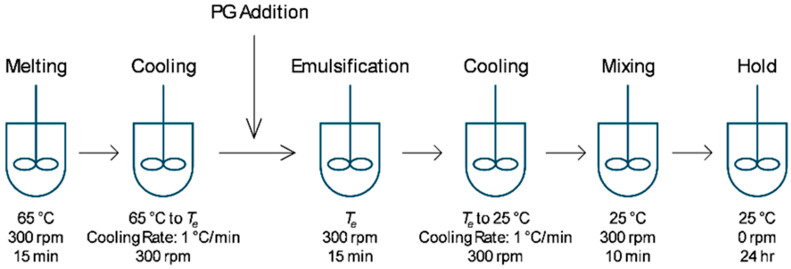
Process flow diagram for emulsion preparation.

Emulsions were prepared with mineral oil as the continuous oil phase, PG as the polar dispersed phase and either MDG, MG or DG as the stabilizing agent. The emulsion composition is summarized in Table 1. Desirable amounts of mineral oil and emulsifying agent were added into a PYREX® 250 ml jacketed vessel (Corning Inc. New York, NY, USA) connected to a JULABO FP50 circulator (JULABO GmbH, Seelbach, Germany) for temperature control. The mixture was heated to 65 °C and stirred using an IKA EUROSTAR digital overhead stirrer equipped with an anchor stirrer (IKA, Staufen, Germany) at 300 rpm for 15 min until all components were fully melted. The vessel was then cooled at a rate of 1 °C/min while mixing at 300 rpm until the emulsification temperature (T_e_) was reached. At T_e_, an appropriate amount of PG was added, and the mixture was maintained at T_e_ while mixing at 300 rpm for 15 min to allow the emulsion to form. The emulsion was then cooled to 25 °C at 1 °C/min and maintained at that temperature for 3 h under mixing at 300 rpm. Finally, the emulsion was held at 25 °C without mixing for 24 h before characterization (Figure 1). The compositions and T_e_ values used for all samples studied here are listed in Table 2.

### 2.3. Isolation of Diglycerides (DG) from Mono-and-Diglyceride (MDG)

A total of 10 wt% of MDG was dissolved in mineral oil at 65 °C. The solution was then cooled to 45 °C and maintained at that temperature under constant stirring for 1 h. At that temperature, MG precipitated, while DG remained dissolved in the mineral oil. The suspension was then centrifuged using an Eppendorf Centrifuge 5810R (Eppendorf, Hamburg, Germany) at 6000 rpm and 40 °C for 20 min. The supernatant containing the dissolved DG was collected and allowed to cool to room temperature (~23 °C). The precipitated DG was then separated through centrifugation at room temperature at 6000 rpm for 20 min. After centrifugation, the solid paste was collected, and excess mineral oil was removed as much as possible using filter paper. 

### 2.4. Characterization

#### 2.4.1. Microscopy Analysis

Microscope images of the emulsions were obtained using an Olympus polarizing BX51 microscope (Olympus, Tokyo, Japan) (PLM) equipped with a Nikon DS-Fi3 high-resolution camera (Nikon, Tokyo, Japan) both in brightfield and polarized light mode. The crystallization behavior of MDG, MG and DG in mineral oil under cooling was monitored using a Linkam THMS600 hot stage connected to a T95 LINK and an LNP 95 cooling system (Linkam Scientific Instruments Ltd., Surrey, UK). A few milligrams of the samples containing different compositions of the solid in mineral oil were loaded into a quartz crucible and mounted onto the hot stage. The sample was heated to 60 °C and maintained at that temperature for 5 min to ensure complete melting. It was then cooled to 25 °C at a cooling rate of 1 °C/min. Time-lapsed images were taken at an interval of 1 min.

The morphology of distilled MG and isolated DG was also checked using polarized microscopy. For distilled MG, the solid was dissolved in mineral oil at 5 wt% and then heated until all solids were melted. The mixture was then cooled to 25 °C at 1 °C/min to allow the MG to crystalize before examination under the microscope. For isolated DG, the crystal morphology was checked using a sample where the isolated DG was diluted with mineral oil 3 times to obtain a dilute solution of DG in mineral oil.

#### 2.4.2. Physical Stability

The physical stability of the different emulsions was characterized using a LUMiSizer (LUM GmbH, Berlin, Germany). The LUMiSizer employs centrifugal forces to accelerate the phase separation in the samples while light transmitted through the samples is measured across the sample length as a function of time and position. An approximately 0.5 ml sample was placed into a polycarbonate cell (LUM GmbH, Berlin, Germany) with an optical path of 2 mm. The samples were subject to centrifugation at either 1000 rpm (for emulsion samples containing neat MG or DG) or 4000 rpm (for emulsion samples containing MDG) at 25 °C. Each sample was measured six times. 

#### 2.4.3. Dynamic Scanning Calorimetry (DSC)

The crystallization temperature (T_c_) of the stabilizing agents was measured using a Mettler Toledo DSC3 calorimeter (Greifensee, Switzerland). Approximately 10 mg of the emulsion sample was placed into a crimped aluminum pan (Mettler Toledo, Greifensee, Switzerland) and heated to 70 °C at 10 °C/min. The sample was then equilibrated at 70 °C for 5 min, before cooling to 25 °C at a rate of 1 °C/min. Each measurement was performed in triplicate. The onset temperature during cooling was recorded as the crystallization temperature.

#### 2.4.4. X-Ray Diffraction (XRD) and Small- and Wide-Angle X-Ray Scattering (SAXS)

The polymorphic form of the stabilizing crystals present in the emulsions was analyzed using a Bruker D8 Advance powder X-ray diffractometer (XRD) (Bruker, Billerica, MA, USA) with CuKα radiation operated at an acceleration voltage of 35 kV and a current of 40 mA. The scans were performed in the range of 3° to 30° 2θ at a step size of 0.01° with a scan rate of 1°/min. Data smoothing and background subtraction were performed using the DIFFRAC.EVA V7 software (Bruker, Billerica, MA, USA).

The polymorphs formed during MDG crystallization from mineral oil were determined by performing SAXS/WAXS measurements at the Elettra Synchrotron facility (Elettra, Trieste, Italy). The incident beam energy was set to 8 keV (1.54 Å wavelength). Calibration was performed using silver behenate (d-spacing = 58.38 Å). The SAXS detector was a Pilatus3 1M, while for the WAXS measurements, a Pilatus 100k detector was used. A total of 10 wt% of MDG in mineral oil was filled into a quartz capillary with a 1.5 mm outer diameter (Hampton Research, Aliso Viejo, CA, USA) and inserted into a KPR (Peltier heating/cooling) sample holder (Anton Paar, Graz, Austria). Initially, the sample was heated to 65 °C and maintained for 5 min to ensure complete melting. The sample was then cooled from 65 °C to 25 °C at a rate of 1 °C/min. The SAXS and WAXS patterns were collected every 1 min during the cooling experiment with an exposure time of 20 s.

#### 2.4.5. Rheology Measurements

The rheological properties of the emulsion samples were analyzed using an MCR 302 rheometer (Anton Paar, Graz, Austria). All measurements were carried out at 25 °C using smooth steel parallel plates with a diameter of 25 mm and a gap of 1 mm. Before each measurement, a rest period of 10 min was allowed to relieve residual stress and facilitate the reformation of the emulsion’s microstructure. Viscoelastic properties, including the storage modulus (G′), loss modulus (G″) and crossover stress (σ_cross_), were derived from amplitude sweep tests at a frequency of 1 Hz, using a strain range from 0.01% to 1000%. Viscosity curves were also measured using a shear thinning test method with the shear rate ranging from 0.001 to 100 s^−1^.

#### 2.4.6. Contact Angle Measurement

The three-phase contact angle formed by PG at the phase boundary of the mineral oil and stabilizing agent (either MDG or MG) was measured using an OCA 25 Contact Angle System (DataPhysics, Filderstadt, Germany). The stabilizing agent (either MDG or MG) was melted and then rapidly cooled to form a flat surface on the bottom of a transparent quartz box, which served as the substrate for contact angle measurements. Mineral oil was then added into the quartz box to create a phase boundary between the mineral oil and the stabilizer. A drop of PG (2 µl) was then placed onto the solid substrate immersed in mineral oil at a flowrate of 0.5 µl/s using a Hamilton Gastight 0.5 ml syringe (Reno, NV, USA) and a precision tip needle (Nordson, Westlake, OH, USA). Contact angle measurements were triplicated for each stabilizing agent used.

## 3. Results

### 3.1. Crystallization Behavior of MDG in Mineral Oil

**Figure 2:**
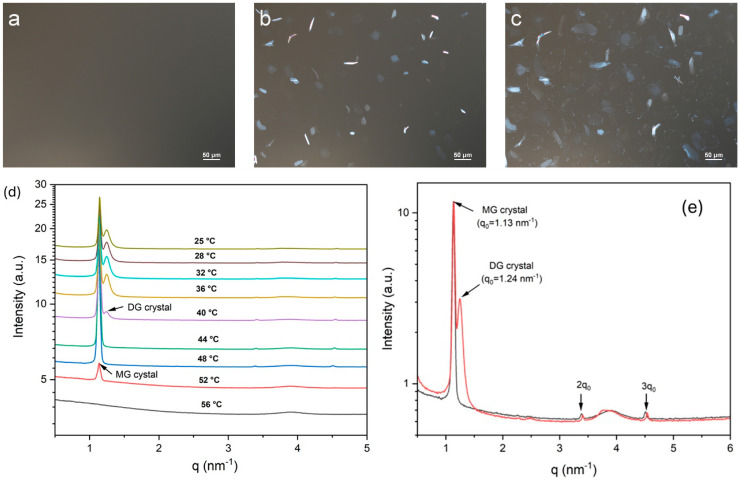
Polarized light microscope images obtained while cooling MDG solution in mineral oil: (**a**) 55 °C, (**b**) 53 °C and (**c**) 40 °C. (**d**) Temperature-resolved SAXS patterns acquired during the cooling crystallization process. (**e**) SAXS patterns acquired at 48 °C (black) and 36 °C (red).

The crystallization behavior of MDG in mineral oil was examined using polarized light microscopy (PLM) and X-ray scattering. For PLM, MDG dissolved in mineral oil at 65 °C was cooled at 1 °C/min using a temperature-controlled hot stage, and time-lapsed images were taken at 1 min intervals. Figure 2a–c show the microscope images of the solution of MDG in mineral oil at 10 wt% during cooling at different temperatures. Initially, at high temperatures, no crystals were observed under PLM (Figure 2a), and later, plate-like crystals were observed at around 53 °C (Figure 2b). Under cross-polarized illumination, the crystals appeared bright and elongated when oriented parallel to the optical axis; on the other hand, the crystals appeared dim when oriented perpendicular to the optical axis. As the solution temperature decreased further, more plate-like crystals were formed. At around 40 °C, a second, distinct population of much smaller crystals appeared (Figure 2c). These crystals appeared to nucleate around the edges of the larger plate-like crystals, indicating secondary nucleation. Previously, Ali et al. [12] observed similar temperature-dependent crystallization behavior for MDG in paraffin oil, except that the second type of crystals exhibited a spherulite morphology. Using ^1^H NMR spectroscopy, the plate-like (crystalized at 50 °C) and spherulite (crystalized at 40°) crystals were identified as MG and DG crystals, respectively [12]. Here, we adopt the same allocation of chemical identity, where plate-like crystals crystalized at 53 °C were identified as MG, and the small crystal population precipitated at 40 °C was identified as DG. 

We used a synchrotron radiation SAXS/WAXS analysis to characterize the structural properties of the plate-like MG and small-sized DG crystals formed during the crystallization of MDG from mineral oil. Temperature-resolved SAXS patterns acquired during the cooling crystallization process are shown in Figure 2d. At high temperatures, no scattering peaks were observed, suggesting complete dissolution of MDG in the mineral oil. A scattering peak at *q* = 1.13 nm^−1^ (*d* = 5.56 nm) appeared when the system was cooled to 52 °C, corresponding to the nucleation of MG crystals. A scattering peak at *q* = 1.24 nm^−1^ (*d* = 5.07 nm) appeared when the temperature decreased to 40 °C, indicating the formation of DG crystals. These observations are consistent with the crystallization behavior of MDG monitored using PLM. The periodic peaks observed in the SAXS patterns for the MG and DG crystals (Figure 2e) confirm the lamellar structure of these crystals.

**Figure 3:**
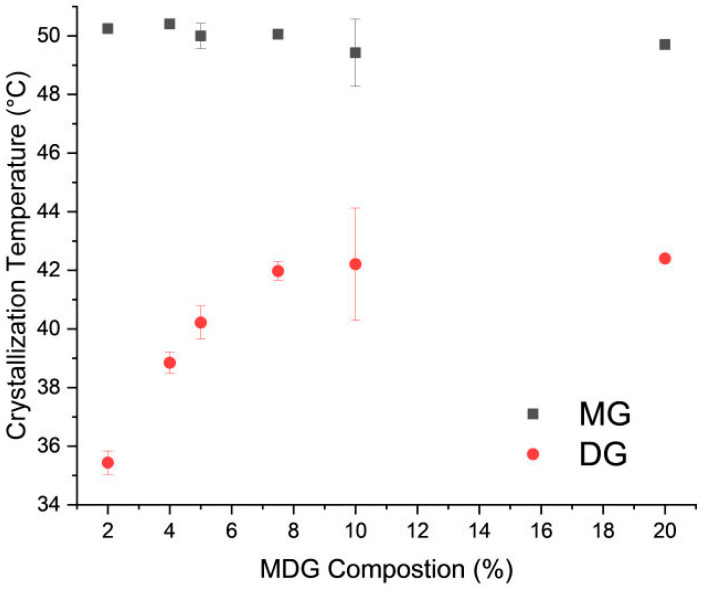
Crystallization temperature of MD and DG crystals as a function of MDG concentration in mineral oil. Error bars are standard deviations, n = 3.

The crystallization temperature of MG and DG at different MDG concentrations was also measured through DSC. Two distinct crystallization peaks—the first and second peaks corresponding to MG and DG crystallization, respectively—can be observed from the DSC cooling thermograms (Supporting Information Figure S1). On varying the concentration of MDG in mineral oil, the crystallization temperature of MG remained constant at around 50 °C, while that of DG initially decreased linearly with concentration and then stabilized (Figure 3). A similar pattern between the DG crystallization temperature and MDG concentration in paraffin oil was reported by Ali et al. [12].

**Figure 4:**
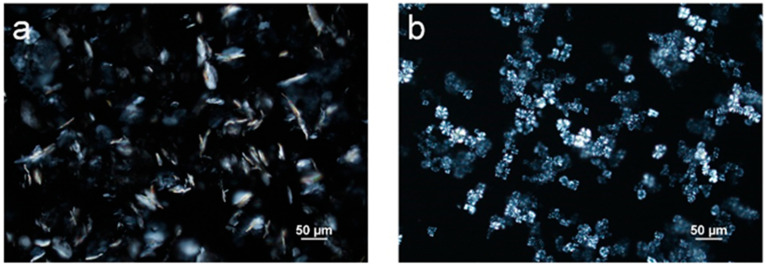
Polarized light microscope images of (**a**) neat monoglycerides (MGs) and (**b**) neat diglycerides (DGs) dissolved in mineral oil at 5 wt%.

In order to study the influence of the crystallization of individual MG and DG on the emulsion properties, neat samples of MG and DG are needed. Since neat DG is not commercially available, it was obtained through separation from MDG, as described in the Materials and Methods section. The samples were inspected with PLM (Figure 4b) to confirm that neat DG was obtained from MDG. In the samples obtained after separation, a single population with a spherulite morphology, characterized by a “Maltese cross” appearance under polarized light, was observed [46]. The nature of the sample was further confirmed through a DSC analysis, where a single crystallization temperature was observed, at 40 °C, suggesting that the sample contained only DG crystals (Supporting Information Figure S2b). Since the separated DG contained a certain amount of mineral oil, which is difficult to remove due to its non-volatile nature, the concentration of DG in the sample had to be determined. Since the crystallization temperature of DG varies with its composition in mineral oil, it could be used to estimate the concentration of DG in the obtained sample. The obtained DG sample was diluted 10 times in order to reach a DG composition that fell within the linear range. The crystallization temperature was then measured using DSC (Supporting Information Figure S2b). The obtained DG sample was estimated to contain 15 wt% of DG.

Commercially available distilled MG samples were purchased and used without further purification. Figure 4a shows a polarized microscopic image of MG crystallized from mineral oil at 5 wt%, and only plate-like crystals were observed for this sample. The crystallization temperature of the purchased MG dissolved in mineral oil at 5 wt% was measured using DSC and was determined to be 54 °C (Supporting Information Figure S2a).

### 3.2. Microstructure of Emulsions Prepared with Different Emulsification Temperatures (T_e_s) and Stabilizers

**Figure 5:**
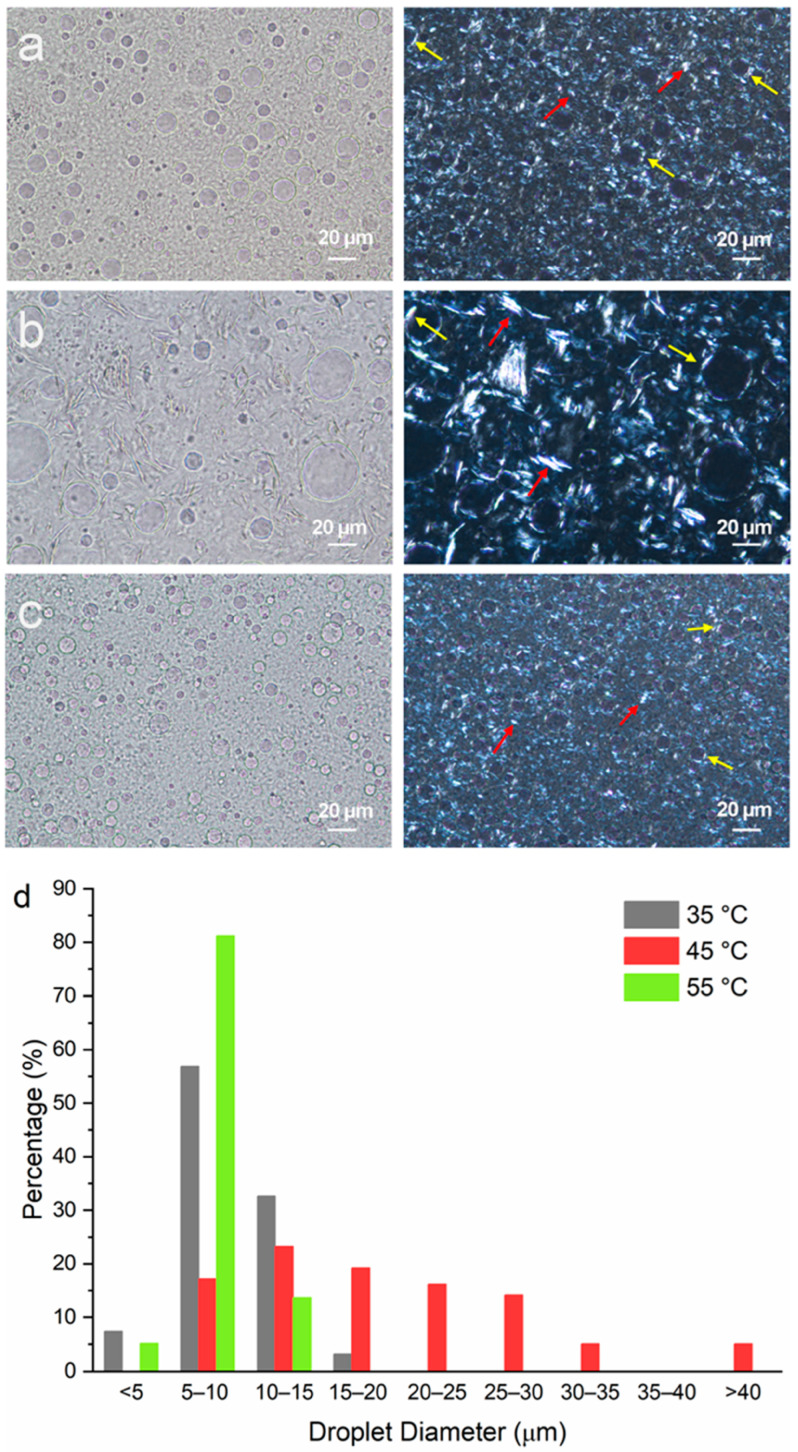
Microscopic images of emulsions containing mineral oil–PG–MDG at a composition of 80–10–10 (by wt%) prepared at emulsification temperatures of (**a**) 35 °C, (**b**) 45 °C and (**c**) 55 °C. Images shown in the left column were taken under brightfield, and the images in the right column were taken under polarized light. Red arrows show plate-like crystals in the bulk oil phase, and yellow arrows show crystals formed at the PG–mineral oil interface. (**d**) The size distribution of the PG droplets in emulsions prepared at different T_e_ values.

Emulsion samples containing mineral oil, PG and MDG at 80, 10 and 10 wt%, respectively, were prepared at different emulsification temperatures. The microscope images of the emulsion samples acquired under brightfield mode show circular PG droplets (Figure 5a–c) with diameters ranging between 5 and 40 µm (Figure 5d). The size distribution of the PG droplets was measured manually from the microscope images using ImageJ software version 1.54. At least 100 droplets were measured to ensure statistical significance. In general, the droplet size is found to be smaller when T_e_ = 35 °C or 55 °C and is much bigger for T_e_ = 45 °C. For T_e_ = 55 °C, the PG droplet sizes predominantly ranged from 5 to 10 µm, with a small percentage of the droplets showing diameters either less than 5 µm or between 10 and 15 µm. For T_e_ = 35 °C, slightly larger droplets are observed, with a decrease in the percentage of droplets between 5 and 10 µm and a simultaneous increase in the number of droplets in the size range of 10 to 15 µm. However, when emulsification was carried out at 45 °C, a much broader size distribution, with droplets of a size larger than 40 µm, was obtained. Equally, in this case, the size of the stabilizing agent crystals was relatively bigger (Figure 5b, right panel).

In a previous study, Al et al. found that MG crystals were preferentially adsorbed at the polar liquid (polyethylene glycol)/paraffin oil interface [12]. However, in this study, the plate-like MG crystals resided both in the bulk oil phase and the polar liquid (PG)/mineral oil interface (Figure 5). This is more apparent in Figure 5b, where large plate-like crystals can be seen to be adsorbed onto the droplet surface (red arrows), as well as in the interstitial space between the droplets (yellow arrows). This observation shows that MG crystals do not show preferential affinity towards the droplet surface, which can be attributed to the different oil phases (mineral oil versus paraffin oil) and polar liquids (PEG versus PG) used in the current versus the previous study [12]. It can be inferred that MDG provided both Pickering and network stabilization to the emulsions. 

Although DG exhibited a spherulite morphology when crystallized from a dilute solution of mineral oil (approximately 5 wt% DG) (Figure 4b), no spherulites were observed in the MDG-stabilized emulsions. This could be attributed to the presence of MG crystals and PG droplets, which in turn could potentially induce secondary nucleation. Also, due to the small size and high density of the crystals present in the emulsion formulation, it was difficult to clearly identify the DG crystals from the PLM images (Figure 5a–c). Therefore, emulsions containing a smaller concentration of MDG (5 wt%) were prepared to observe the distribution of the DG crystals better (Supporting Information Figure S3). Interestingly, the characteristic spherulite morphology of the DG crystals was only observed in the emulsion prepared at an emulsification temperature of 35 °C. This could be a result of primary nucleation of DG in mineral oil, without the influence of the droplet surfaces, prior to emulsification at 35 °C. On the other hand, for T_e_ = 45 or 55 °C, the droplets were generated prior to DG crystallization, which then influenced the nucleation and crystal growth behavior. At the early stage of spherulite formation, they often appear as sheaves of plate-like crystals [47,48], before developing into full spherulites. When DG crystallization occurred after emulsification, the presence of phase boundaries in the system may have potentially inhibited the growth of the DG crystals into full spherulites. For the emulsion where DG spherulites were observed (at T_e_ = 35 °C) (Figure S3a), they resided mainly in the bulk oil phase, and no DG spherulites were observed at the phase boundary. This can be attributed to the hydrophobic nature of DG crystals. In line with this notion, it has been reported that in o/w emulsions stabilized using spherulite starch, the starch spherulites predominantly remained in the bulk aqueous phase instead of at the o/w interface due to their highly hydrophilic nature [49].

**Figure 6:**
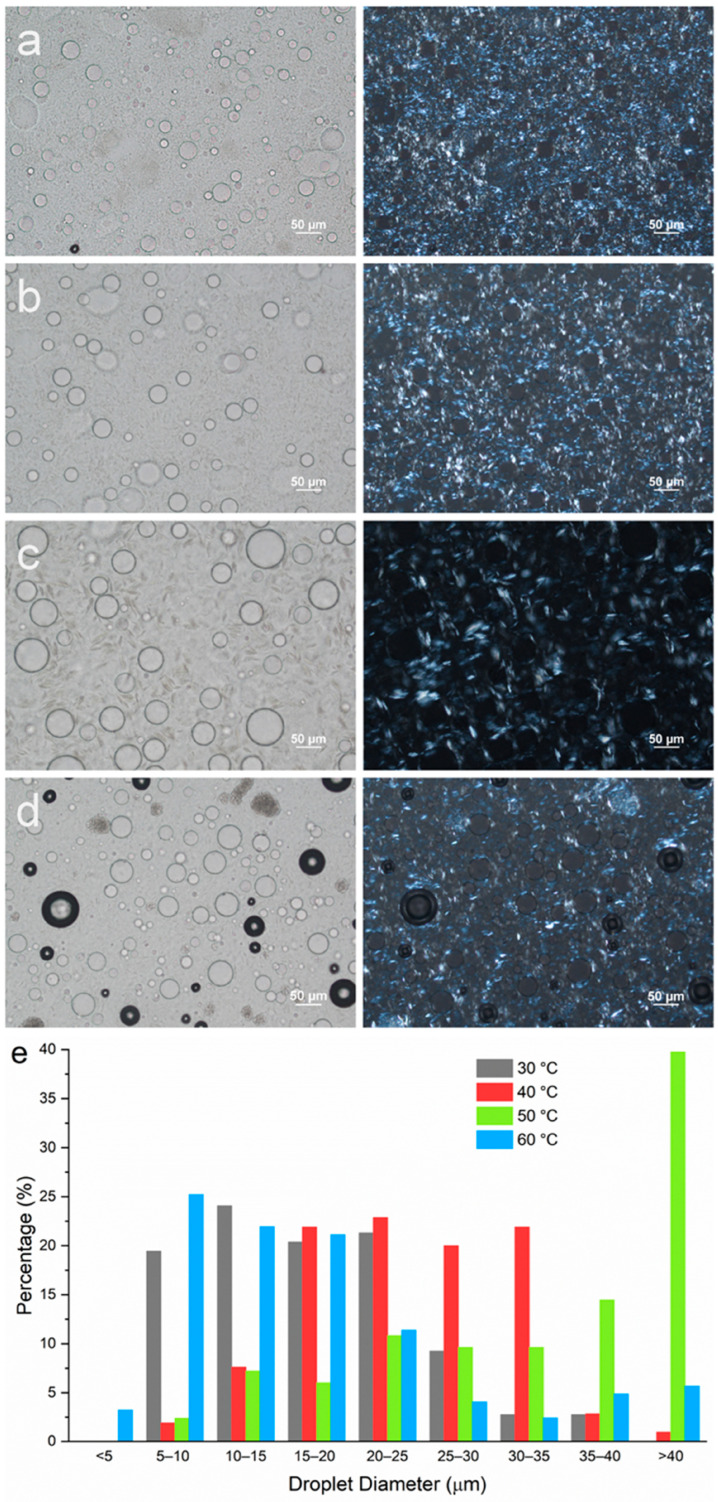
Microscopic images of emulsions containing mineral oil–PG–MG at a composition of 80–10–5 wt% with emulsification temperatures of (**a**) 30 °C, (**b**) 40 °C, (**c**) 50 °C and (**d**) 60 °C. Images shown in the left column were taken under brightfield, and images in the right column were taken under polarized light. (**e**) Size distribution of PG droplets in emulsions with different T_e_ values.

To understand the individual effects of MG and DG, emulsions containing either MG or DG as the stabilizing agent were prepared at different emulsification temperatures. The composition used was mineral oil–PG–MG/DG at 80–10–5 wt%. A stabilizing agent level of 5 wt% was selected since the amount of each individual component in the original formulation containing MDG at 10 wt% was about 5 wt% based on the MDG product specifications. Figure 6a–d show the microscopic images of emulsions containing MG only prepared at different T_e_ values. Since neat MG has a crystallization temperature of 54 °C at a 5 wt% concentration, an emulsification temperature of 60 °C was selected to represent the post-crystallization situation where emulsification occurs before crystallization. Emulsions with T_e_ = 50, 40 and 30 °C were prepared to represent the pre-crystallization procedures (emulsification occurs after crystallization). The size distribution of the PG droplets for emulsions containing only MG as the stabilizing agent is summarized in Figure 6e. The size distribution of the PG droplets at T_e_ = 60 °C (post-crystallization) is similar to that at T_e_ = 30 °C (pre-crystallization), where the majority of the droplets are in the size range of 5–25 µm. When T_e_ = 40 °C, the droplet size distribution slightly shifted to the right, to 15–35 µm. PG droplets with the largest size are observed when T_e_ = 50 °C, with a significant proportion of the droplets above 40 µm. 

For all emulsion samples, similar to the MDG-stabilized emulsions, plate-like crystals could be observed both at the droplet interface and in the bulk oil phase, suggesting that MG crystals do not preferentially reside in either the continuous phases or at the phase boundaries for PG/mineral oil systems. However, as the emulsification temperature changed, the size of the MG crystals formed was affected significantly. The largest crystals were observed when emulsification was carried out at 50 °C, which was just after MG crystallization occurred. Crystal size decreased as emulsification temperature was lowered further below the crystallization temperature. On the other hand, smaller MG crystals were also obtained for emulsification at 60 °C (post-crystallization). The trend in the MG crystal size also followed the trend in the PG droplet size, where smaller MG crystals were observed in emulsion samples with smaller PG droplet sizes.

**Figure 7:**
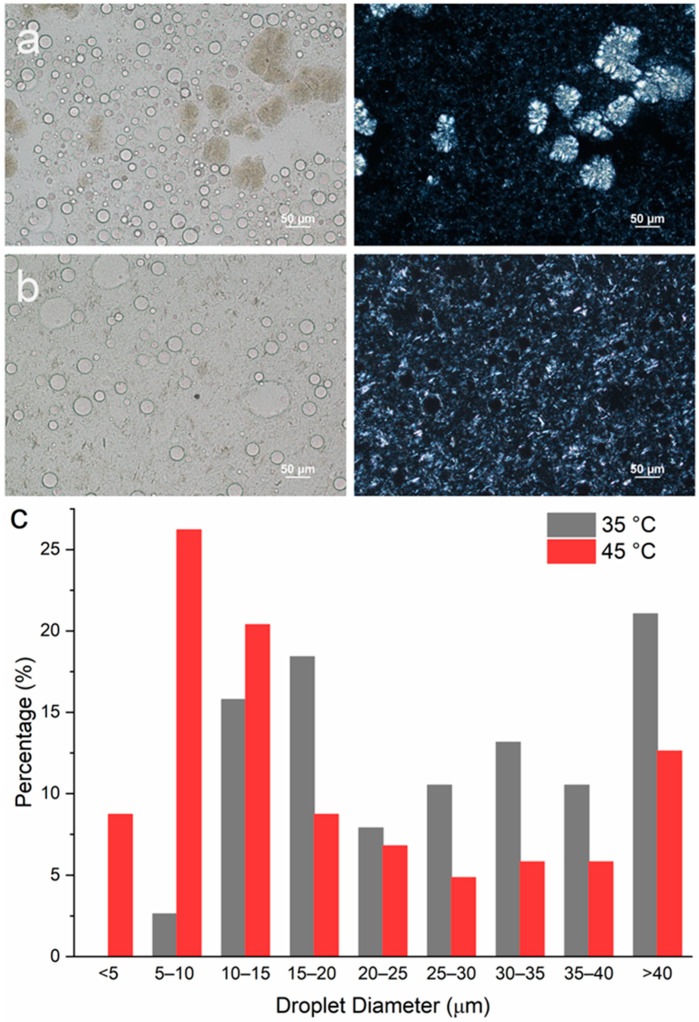
Microscopic images of w/o emulsions containing mineral oil–PG–DG at a composition of 80–10–5 with emulsification temperature of (**a**) 35 °C and (**b**) 45 °C after preparation. The images in the left column were taken under brightfield while the images in the right column were taken under polarized light. (**c**) Size distribution of PG droplets in emulsions prepared at different T_e_s.

To study the impact of emulsification temperature on emulsion microstructures containing DG only, two samples with T_e_ = 35 and 45 °C were prepared, representing a situation where emulsification occurred after and before crystallization, respectively. At a 5 wt% DG concentration, both emulsions underwent phase separation during the 24 h holding period, and no stable emulsions could be prepared. Therefore, a higher concentration of DG (10 wt%) was used. When emulsification was carried out at 35 °C, a significant amount of DG crystals exhibited the spherulite morphology, where Maltese crosses were observed from the PLM images (Figure 7a). However, in the emulsion sample prepared at T_e_ = 45 °C, no spherulites were formed. Rather, sheaves of plate-like crystals were observed (Figure 7b). This observation agrees with the emulsion samples stabilized with MDG (Figure S3). The droplet size distribution is indicated in Figure 7c, where in general, smaller PG droplets were formed when T_e_ = 45 °C as compared to those at T_e_ = 35 °C. 

### 3.3. Stability of Emulsions Prepared at Different Temperatures (T_e_s)

**Figure 8:**
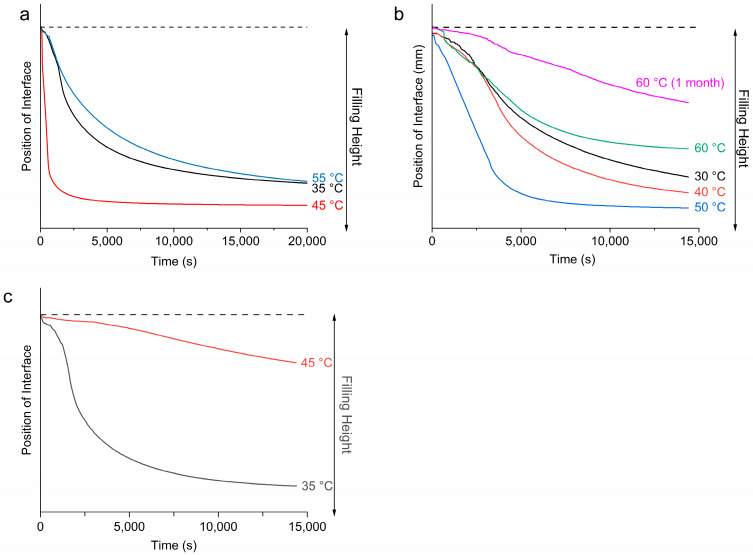
Movement of oil–solid phase boundary when emulsions were subject to centrifugal force in the LUMisizer for emulsions containing (**a**) 10 wt% of MDG (at 4000 rpm), (**b**) 5 wt% of MG (at 1000 rpm) and (**c**) 10 wt% of DG (at 1000 rpm).

During the LUMiSizer stability analysis, emulsion samples were subjected to centrifugal force, which induced phase separation of the samples due to their inherent thermodynamic instability [24,25,50]. As the emulsions underwent phase separation, two layers were formed: a clear liquid layer on top and a turbid layer at the bottom. Since mineral oil has a lower density than propylene glycol, it is expected that the top liquid layer contains largely mineral oil, and the bottom turbid layer consists of the denser PG droplets. The movement of the boundary between the top and bottom layers with time can be tracked and plotted to indicate the rate of phase separation [51]. The movement of this phase boundary is plotted in Figure 8a–c. The top dotted line represents the boundary between air and the emulsion, which remained relatively unchanged throughout the measurement. The solid lines represent the movement of the boundary between the top and bottom layers, which moved downwards with time as the samples were subjected to centrifugal force, suggesting an increasing extent of bleeding. 

For the emulsion samples stabilized by 10 wt% of MDG, the most unstable sample with the highest rate of phase separation was when T_e_ = 45 °C, and the extent of bleeding was also the largest. This corresponds to emulsification occurring before the crystallization of MG crystals but after the crystallization of DG crystals (post-crystallization into MG and pre- into DG). The most stable emulsion was formed when T_e_ = 55 °C, for which both the rate of phase separation and the extent of bleeding were the smallest. This corresponds to emulsification occurring before the crystallization of both MG and DG crystals (post-crystallization into both MG and DG). When T_e_ = 35 °C, the rate of phase separation was slightly faster, whereas the extent of bleeding was similar to that at 55 °C (pre-crystallization into both MG and DG). In this case, emulsification occurred after crystallization of both types of crystals.

Figure 8b shows the movement of the phase boundary of the emulsions containing 5 wt % of MG as the stabilizing agent. At this concentration, the crystallization temperature of the MG was around 54 °C. From Figure 8b, the most stable emulsion was formed when emulsification was carried out at 60 °C (post-crystallization), with the smallest extent of bleeding and the slowest rate of phase separation. This supports our visual observation that among the four MG-containing emulsion samples, only the one prepared at T_e_ = 60 °C did not exhibit phase separation during storage of 1 month. For the other three pre-crystallized samples, where emulsification was carried out after MG had crystallized, the emulsion stability increased as the emulsification temperature decreased, with the most stable emulsion formed at T_e_ = 30 °C, followed by 40 °C and then 50 °C. This observation suggests that higher undercooling led to more stable emulsions. Surprisingly, in the case of the emulsion prepared at T_e_ = 60 °C and stored for 1 month, the emulsion became more stable over time (Figure 8b). Figure 8c shows the movement of the phase boundary for emulsions containing only DG (10 wt%) as the stabilizing agent. A more stable emulsion was formed when T_e_ = 45 °C compared to T_e_ = 35 °C. That is, a more stable emulsion was formed when emulsification occurred before the crystallization of DG crystals. 

The stability of the emulsions can also be inferred from the instability indices (Table 3), where a smaller instability index indicates a more stable emulsion. Note that the instability indices shown in Table 3. were measured under a centrifugation speed of 4000 rpm for the emulsions containing MDG and 1000 rpm for the emulsions containing either MG or DG. Therefore, the instability index cannot be used to compare between emulsions with different stabilizing agents. However, the emulsion stability with different stabilizing agents can be inferred from visual observation of the phase separation upon storage. Emulsions containing MDG are in general more stable compared to emulsions containing only MG or DG, as no phase separation was observed for all T_e_ tested, which suggests that both components of MDG indeed contribute to emulsion stability. Between MG and DG, MG was a more efficient stabilizing agent for this PG in the mineral oil system, as no stable emulsions could be prepared using neat DG at 5 wt%. For the emulsions that remained stable within 1 month of storage, the instability index was measured again to track any changes in emulsion stability. For all of the MDG-stabilized samples, no significant differences in stability were observed. However, for the MG-stabilized emulsion at T_e_ = 60 °C, a significantly smaller instability index was observed, suggesting that the sample became more stable upon storage. 

### 3.4. Rheological Properties

Oscillatory rheology measurements were performed to study the viscoelastic behavior of the emulsion samples. Viscosity curves were also measured and are shown in Supporting Information Figure S4. All emulsion samples showed non-Newtonian and shear thinning behavior. The crossover stress of all of the emulsion samples analyzed in this study is summarized in Table 4. In general, the oscillatory rheology measurements suggested that all emulsion samples were viscoelastic, with higher G′ higher than G″ under very small strain (a representative graph for oscillatory measurement is shown in Figure S5), indicating solid-like behavior. As the strain/stress increased beyond a critical point, both G′ and G″ decreased until they intersected at the crossover stress. Beyond this point, G′ fell below G″, signifying a transition from solid-like to liquid-like behavior and disruption of the emulsion’s internal structure. The strain/stress range over which G′ and G″ remained constant defines the linear viscoelastic region (LVR) [52]. For our samples, the LVRs were relatively narrow. This can explain the shear thinning behavior observed in the viscosity curve as the shear rate used in flow measurements was in the non-linear viscoelastic region. For all emulsion samples measured here, the trend in the crossover stress followed that for stability, where a higher crossover stress translated into a more stable emulsion. Oscillatory rheology measurements were also performed after 1 month of storage for samples that remained stable during that period. For the MDG-stabilized emulsions, a slight increase in crossover stress was observed, whereas the increase was much more significant for the MG-stabilized sample at T_e_ = 60 °C. The viscosity of this emulsion sample also increased significantly over the storage period (Figure S4c). The changes in rheology agreed with the increase in stability upon storage for this emulsion sample as well.

### 3.5. X-Ray Diffraction (XRD)

**Figure 9:**
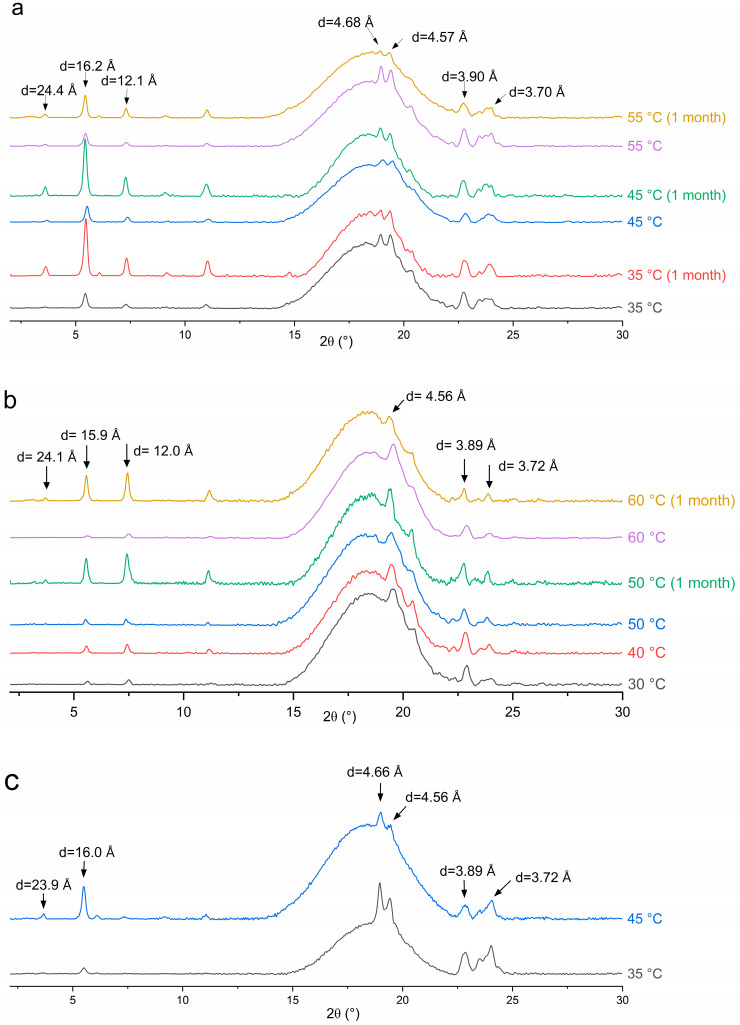
XRD pattern for emulsion samples stabilized with (**a**) MDG, (**b**) MG and (**c**) DG.

XRD diffractograms of the emulsion samples after preparation and storage for 1 month are shown in Figure 9. For the emulsion sample containing MG and prepared at T_e_ = 50 °C, the dense phase at the bottom was used for XRD measurement after storage since the sample underwent phase separation. The broad scattering peaks of all diffractograms were due to the presence of large amounts of mineral oil in the emulsion samples.

For all three stabilizing agents, diffraction peaks at similar angles were present. The small-angle peaks at around d = 24.0, 16.0 and 12.0 Å correspond to the ½, ⅓ and ¼ long spacing of the lamellar glyceride bilayer, with the lamellar thickness determined to be about 48 Å [33]. The appearance of diffraction peaks at wide angles with *d*-spacing in the range of 4.50 to 4.70 Å and 3.70 to 3.90 Å suggests that the glyceride crystals are most likely in the stable β crystalline phase [33,35]. The characteristic peak for the metastable α-gel phase at around *d* = 4.17 Å [33] was not observed in the XRD patterns. Therefore, glyceride crystals did not exist in the metastable α-gel phase after processing. The *d*-spacing values for the lamellar bilayer measured in the emulsion samples using XRD differed slightly from that measured in solutions using SAXS (5.07 and 5.56 nm), suggesting the formation of other polymorphs, such as an inverse lamellar phase, while cooling the MDG solution in the capillaries during the SAXS analysis [33]. During emulsion preparation, the stabilizing agent crystals are subjected to significant mechanical shear and a long holding period of 24 h. It is possible that the metastable glyceride crystal phases transformed into the stable β polymorph during processing or the holding period [35]. 

However, while the peak positions are the same across all emulsion samples, the relative peak intensity between long spacing and short spacing peaks varies with the stabilizer used or the emulsification temperature. For example, the relative intensity ratio between the small-angle and wide-angle diffraction peaks was higher when T_e_ = 45 °C compared to that when T_e_ = 35 °C for the DG-stabilized emulsions (Figure 9c). This can be explained by the differences in crystal morphology and size, despite them having the same polymorphic form. Here, DG crystals exist as large spherulites when T_e_ = 35 °C and as sheaves of plate-like crystals when T_e_ = 45 °C (Figure 7a,b). Crystal morphology can affect the relative peak intensities, as it affects how X-rays diffract from crystal faces [53]. For the MG-containing emulsions, the ratio of the peak intensity remained similar for different T_e_s. This is consistent with the observations that the MG crystals exhibited the same plate-like morphology across different T_e_s, with variations observed only in the crystal size (Figure 6).

After the 1-month storage, no shift in the peak position was observed for any sample, suggesting that no polymorphic transition occurred. This is expected, as the β crystalline form is the most stable form of glyceride in a hydrophobic environment. However, the relative peak intensity was altered after storage. For the emulsions containing MG or MDG, the small-angle peaks characteristic of the lamellar bilayers became significantly more intense relative to the wide-angle peaks upon storage. The PLM images indicate that while there were no significant changes in the crystal morphology upon storage (Figure S6), and the crystal size increased over time, potentially leading to changes in the relative intensity of different peaks. However, an increased crystal size cannot explain the increased stability and strengthening of the crystal network after storage, as an increasing crystal size usually leads to weaker structures. Lin et al. [54] suggested that crystal networks are hierarchical structures consisting of primary crystal elements that aggregate into intermediate crystal clusters, which then further aggregate into crystal microstructures. Here, restructuring of the crystal network at different hierarchical levels may have been responsible for the strengthened networks, while the crystal polymorph and morphology remained the same.

### 3.6. Contact Angle

Representative images of the three-phase contact angle (θ) of PG on the solid substrate immersed in mineral oil are shown in Table 5. The value of the three-phase contact angle (θ) depends on the relative strength of three interfacial tensions (γ), γ_op_ (the interfacial tension at oil–PG interface), γ_os_ (the interfacial tension at the oil–substrate interface) and γ_ps_ (the interfacial tension at the PG–substrate interface) [55]. It is a key parameter that dictates the adsorption of the solid at the oil–PG interface in emulsion systems, where the free energy of attachment $\left(E_{a}\right)$ for a solid spherical particle with the radius *r* can be calculated from the equation below [56]:

(1) $$E_{a}=-\pi r^{2}\gamma_{op}{(1\pm \cos\theta )}^{2}$$

where the + sign in the bracket is used for attachment from the oil phase and the − sign for attachment from the water phase.

According to the equation, the particles are more strongly attached to the interface when *θ* is closer to 90°. Based on the three-phase contact angles, both substrates have *θ* values greater than 90°, where MG has a smaller *θ* value than that of MDG, suggesting that MG crystals are attached at the interface more strongly compared to MDG while both crystals interact with the mineral oil more favorably than with PG [55]. The contact angle of DG could not be measured due to difficulty obtaining a mineral-oil-free DG sample. However, the affinity of DG towards each phase can be inferred from the contact angle of MDG and MG. Since MDG is a mixture of MG and DG, its contact angle is expected to be somewhere between that of MG and neat DG. Therefore, DG is expected to have a contact angle that is higher than that of MDG, suggesting that DG would have an even higher affinity towards the oil phase compared to that of MG or MDG. This is expected, as DG contains two long hydrocarbons chains from the fatty acids, whereas MG contains only one hydrophobic chain. 

## 4. Discussion

### 4.1. Microstructure and Physical Properties of Emulsion Samples

In this study, the microstructure of the emulsion samples stabilized with different stabilizing agents was studied using microscopy. For all three stabilizing agents used, crystals were observed both at the droplet interface, providing Pickering stabilization, and in the bulk oil phase, providing network stabilization. This observation varies from a previous study by Ali et al. [12], where MG formed a shell around polyethylene glycol (PEG) droplets dispersed in paraffin oil whereas DG formed a percolating network in the continuous phase. Such discrepancies can be explained by the different hydrophobic and hydrophilic phases used. It has been reported that the network structure formed by MG in bulk oil is sensitive to the type of oil used since the network is mainly based on intermolecular hydrogen bonding [57]. In addition, the polar liquid used was PEG, which has a different affinity towards either MG or DG, leading to a different distribution of stabilizer crystals in the emulsion samples. 

It is demonstrated here that there is a strong correlation between the microstructure and the physical properties of emulsion samples, including rheology and physical stability. In general, a smaller size of the stabilizing crystals is often accompanied by a smaller droplet size, which then translates into better stability and higher viscosity and crossover stress. It is widely recognized that a smaller droplet size often corresponds to more stable emulsion systems [14,15,16,17,18]. Possible reasons include thermodynamically unfavorable creaming/sedimentation [16,58], better coverage and a larger surface area available for the surfactant at the interface [59] and increased overall viscosity [60]. Many studies have also reported similar correlations between a smaller crystal size and physical stability. For example, for crystals that adsorb onto a droplet interface, small crystals are more effective Pickering particles, as they adsorb more easily at curved surfaces [61,62]. For crystals that provide network stabilization, smaller crystals means a higher network connectivity, which more effectively congeals the bulk oil phase, leading to more stable emulsions, as well as more solid-like structures, reflected by higher rheological parameters [12].

Other than the size of crystals, their morphology also affects physical stability. This can be deduced from the case of DG-stabilized emulsions. When the DG crystals adopted a spherulite morphology, the emulsion sample became less stable, with a less rigid structure. The crossover stress and apparent viscosity were also lower compared to those for the emulsions without DG crystals as spherulites. It is difficult for large spherulites to adsorb onto droplet interfaces, and they mainly resided in the bulk continuous oil phase (Figure 7a), thus forming the part of the crystal network that provided network stabilization to the system. However, the crystal network formed by large spherulites tends to be weak [12]. Other than forming a stronger network, small DG crystals/sheaves of plate-like crystals can adsorb more easily onto the PG droplet interface as well (Figure 7a,b), providing Pickering stabilization. Therefore, by breaking up large spherulites into smaller crystals, PG droplets can be encapsulated better, providing an additional stabilization effect. Similar effects were observed by Metilli et al. [63] and Tenorio-Garcia et al. [64], where breaking up large cocoa butter spherulites allowed the smaller cocoa butter crystals to stabilize the dispersed phase droplets via a Pickering mechanism. The poor stabilizing effect of spherulites has also been reported before. Gao et al. reported that unstable w/o emulsions were formed when additives promoted the formation of large spherulites (more than 100 µm) of anhydrous milk fat, which served as the stabilizing agent. It was also suggested that protruding crystals on the surface of larger spherulites could pierce the water–oil interface, causing coalescence and the formation of larger water droplets [65]. Ghosh and Rousseau [66] reported that spherulites in the bulk continuous oil phase were insufficient to stabilize w/o emulsions, and an increased sedimentation rate was observed. It is noteworthy that several other studies have shown that spherulites, when capable of interacting at the droplet interface, can function as Pickering particles, stabilizing emulsions effectively [13,49,67]. However, such a Pickering mechanism is very much dependent on the chemistry of the system involved. In the system studied here, DG was shown to have poor affinity for the dispersed PG phase, and no spherulites were seen at the droplet interface. Instead, only small sheaves of plate-like crystals were capable of encapsulating the PG droplets (Figure 7a).

### 4.2. Effect of Emulsification Temperature on Emulsion Stability

In this study, it was demonstrated that the temperatures at which emulsification (T_e_) was carried out significantly affected the microstructure of the emulsion samples, and by extension, the physical properties, including rheology and physical stability, for all of the stabilizing agents used. However, the relationship between T_e_ and various physicochemical properties is not a linear one. Rather, it is dependent on the sequence of emulsification and crystallization. For all three stabilizing agents used, when emulsification was carried out before crystallization of the stabilizing agents (post-crystallization), the emulsions formed tended to be more stable, with higher rheological properties compared to the properties in those processed using pre-crystallization procedures (emulsification carried out after crystallization). Various studies have shown that the sequence of emulsification and crystallization affects the microstructure and physicochemical properties of emulsions. In general, solid particles that are created after emulsification followed by cooling (post-crystallization) display better stabilization effects than those of solid particles added or crystallized prior to emulsification (pre-crystallization) [10,68,69]. Different reasons can be proposed for the superior stabilizing effects of the post-crystallization process. 

#### 4.2.1. Crystal Size Reduction

Comparing Figure 6c and d for the MG-stabilized emulsions, significantly larger crystals were formed when emulsification was carried out right after crystallization. As discussed earlier, smaller particles can either be adsorbed more effectively at droplet interfaces or form a more interconnected crystal network in the continuous oil phase, depending on their surface activity, both of which lead to better stability. Such differences in crystal size could possibly be caused by the presence or absence of PG droplets when crystallization occurs. In the post-crystallization regime, PG droplets present in the system act as heterogeneous nucleants for glyceride crystals, thus increasing the nucleation rate of the crystallizing systems. Various mechanisms have been proposed for the increased nucleation rate, including surface adsorption, lattice matching and functional group matching [70,71,72]. With a higher nucleation rate, smaller crystals are more likely to be formed.

For pre-crystallized emulsions, the adverse effect on stability can be partially mitigated by reducing the emulsification temperature. For the emulsions stabilized with either MDG or MG, the most stable pre-crystalized emulsions were formed with the lowest Te (Table 3). The extended mixing time prior to emulsification may reduce the crystal size through shear, thereby contributing to the formation of more stable emulsions. Similar behavior has been reported by Prodromidis et al., where increased stability was achieved using a lower emulsification temperature in the pre-crystallized regime for w/o emulsions stabilized with MG [73]. However, performing emulsification at lower temperatures requires a greater energy input to achieve complete emulsification, which could be a concern for industrial-scale manufacturing. 

#### 4.2.2. Crystal Morphology

For the DG-stabilized emulsions, a different crystal morphology instead of a size reduction was responsible for the better stability of the post-crystallized emulsions. As explained earlier, the formation of large spherulites in the pre-crystallized emulsions hindered physical stability, as they were not able to act as Pickering particles or form strong crystal networks. The formation of large spherulites may potentially have been inhibited when crystallization occurred in the presence of a large amount of phase boundaries. When spherulites start to form, they very often appear as sheaves of plate-like crystals [47,48] before they can develop further into full spherulites. When a large amount of phase boundaries is present in the system, the growth of sheaves of plate-like crystals into full spherulites could be hindered. In contrast, in the pre-crystallization process, DG crystallization takes place in a hydrophobic environment free from any interfaces or templates, thereby facilitating their growth into large spherulites. The subsequent emulsification process may not generate sufficient mechanical shear to break up all of the spherulites formed, resulting in their retention in the final emulsion. It is worth noting that another study reported no significant differences in microstructure or emulsification efficiency between the two procedures [74]. This was attributed to the low crystallinity of the stabilizing agent used, which made it less sensitive to the crystallization conditions. Therefore, the processing conditions should be selected carefully based on the specific systems. 

#### 4.2.3. Spatial Distribution of Stabilizing Crystals

Also, when crystals are formed in the presence of interfaces (post-crystallization), crystallization tends to occur more preferentially at the interface, leading to a better-fitting shell of crystals around the droplets. Li et al. [75] observed that medium- and long-chain diacylglycerol (MLCD) was capable of forming interfacial crystal shells in post-crystallized w/o emulsions, whereas in the pre-crystallized emulsions, MLCD crystals were only found in the continuous oil phase. However, this does not appear to be the case for MDG- or MG-stabilized emulsions: no significant changes in the distribution of the stabilizing agent crystals were observed, where stabilizing agent crystals were found to reside both at the interface and in the continuous oil phase following both procedures. For these two stabilizing agents, the crystal size reduction played a more significant role in enhancing the stability of the emulsion systems under study. 

#### 4.2.4. Droplet Size Reduction

Emulsification temperatures also affected the droplet size significantly, which in turn changed the physical stability of the final formulations. Many previous studies have shown that droplet size in emulsion samples follows complicated correlations with various processing parameters, including the shear applied, mixer geometry, the mode of operation and the viscosity of either phase [59,76,77,78]. Several studies have developed models that relate droplet size to these parameters [79,80], and temperature is known to play a complex role in droplet size [81]. Some studies have suggested that higher temperatures in general produce a smaller dispersed phase [82]. At higher temperatures, the viscosity of both phases and the interfacial tension are lower, resulting in reduced thermodynamic energy needed for emulsification, as shown by Jafari, et al. [83]. This can explain our observation that smaller droplets formed when emulsification was carried out at higher temperatures in the post-crystallization regime. However, with the pre-crystallization procedures, by comparing the MDG-stabilized emulsions at T_e_ = 35 and 45 °C or the MG-stabilized emulsions at T_e_ = 30, 40 and 50 °C, a dispersed droplet size is found to decrease with decreasing T_e_. This could be attributed to an increase in viscosity as the temperature decreases. Several studies have shown that for w/o emulsions, an increase in continuous phase viscosity leads to a decrease in the droplet size for viscous emulsions, as droplet breakup is a result of the sub-eddy viscous stress created in the turbulence [59,84]. In addition, a higher continuous phase viscosity reduces the recoalescence of droplets through retardation of the liquid drainage between droplets [83]. In this study, as T_e_ decreases, the viscosity of the continuous phase increases due to a lower temperature and a higher number of smaller stabilizing agent crystals in the bulk oil phase. This may explain the decreased droplet size as T_e_ decreases for pre-crystallized emulsions. 

### 4.3. Effects of Type of Stabilizing Agent on Emulsion Stability 

In this study, the mechanism of stabilization using commercial MDG was investigated by looking at the individual contributions of the two main components in MDG. Comparing the emulsion stability formed with either MG or DG, MG in general was more efficient in stabilizing a polar liquid-in-oil emulsion system, as no stable emulsions were formed with neat DG at 5 wt% (phase separation occurred after the holding period), whereas stable emulsions could be prepared at the same concentration using neat MG. This could be explained by their different crystal morphologies and affinity towards droplet interfaces. From contact angle measurement, it was inferred that DG has a higher affinity towards the bulk oil phase compared to that of MG. As a result, DG tends to remain in the continuous oil phase, contributing to network stabilization more readily than MG under the same conditions, even though both stabilizing mechanisms are in effect for all systems tested. Furthermore, under certain conditions, DG forms large spherulites that can weaken the network structure further. Taken together, these factors suggest that MG is a more effective stabilizing agent for this particular emulsion system. 

Ali et al. [12] also reported the better stabilizing efficiency of MG compared to that of DG in a PEG-in-paraffin oil system, albeit for different reasons. It was reported that the MG and DG crystals showed very different crystal distributions in the emulsion system. In the PEG-in-paraffin oil system, MG with a plate-like morphology was found to act mainly as Pickering particles, whereas DG with a spherulite morphology formed a network only. The different crystal distribution in different emulsion systems can be attributed to the different chemistry of the systems. The polar liquid phase used in this study (PG) is more hydrophilic than the PEG 400 used by Ali el. [12]. There is also a certain degree of structural similarity between PG and glycerides since glycerides are esters formed from PG and fatty acids. These factors can affect the affinity of the glyceride crystals towards each phase and thus the crystal distribution in the final emulsion products. Therefore, it is important to carefully select the stabilizing agent based on specific systems.

### 4.4. Increased Stability of MG-Stabilized Emulsions upon Storage

An interesting observation was made for the MG-stabilized emulsions in this study, where the stability and rheological properties, including crossover stress and viscosity, increased during storage. In contrast to many previous reports where the emulsifying efficiency of MG reduced over time [32,33], in this case, MG-stabilized emulsions were found to strengthen over time. The reduction in the emulsifying ability over time has been associated with a polymorphic transition from the metastable sub-α phase (α-gel), which is able to retain more oil in its lamellar bilayers, to the stable β crystalline phase [33] and the transition from a smooth droplet surface to a porous one when the polymorphic transition occurs [85]. However, based on the XRD patterns of the emulsion samples before and after storage, no polymorphic transition was observed. The MG crystals in the freshly prepared samples were already in the stable polymorphic form, and a peak shift was not observed before or after storage. 

Strengthening of the structure for MG in oil systems has in fact been observed before. Batte reported an increase in the storage modulus (G′) and melting temperature with time for an emulsion formed with water, canola oil and monostearin [86]. In contrast to the system studied here, the system was an o/w emulsion with a monostearin wall surrounding the oil droplets. Similarly, Ojijo et al. reported an increase in hardness and viscosity during storage for an MG/olive oil gel network [87], and the authors attributed their observation to a decrease in the crystal cluster size and reorganization into a more compact network during storage or sintering. Sintering is a process where solid bridges form within the fat crystal networks, enhancing their strengths [88,89]. In this study, however, crystal size was observed to increase over time from the PLM images. The hypothesis that crystal network reorganization or sintering occurred without a polymorphic transition could explain the strengthening of the crystal network over time. 

Notably, network reorganization and sintering were also evident in the samples that underwent phase separation, as indicated by changes in the relative XRD peak intensities in the bottom layer after storage. These findings suggest that two competing kinetic processes, phase separation via sedimentation and microstructural strengthening, occur during storage. In emulsions that are initially unstable, sedimentation proceeds more rapidly than network strengthening, resulting in macroscopic phase separation. Conversely, emulsions with greater initial stability exhibit a self-hardening effect over time, which contributes positively to their long-term physical stability.

For the samples containing MDG, strengthening of the structure was observed as the crossover stress increased after storage. This is consistent with the presence of MG crystals in MDG which might have undergone similar restructuring that led to strengthening of the crystal network.

### 4.5. Implications for Topical Formulations and Limitations of the Current Study 

Designing formulations and manufacturing processes that yield products with desirable attributes is essential for the development of new drug products. From a formulation perspective, better stabilization effects can be achieved when the stabilizing agent interacts effectively at the droplet boundaries. Therefore, selecting stabilizing agents suited to specific systems is crucial for emulsion-based topical formulations. Three-angle contact measurements can serve as a preliminary screening method to assess how the selected emulsifying agent interacts with each phase. From a processing perspective, the post-crystallization regime provided greater stability in this study. Selecting an appropriate emulsifying temperature based on the crystallization behavior of the stabilizers allows optimized manufacturing processes to be designed. The post-crystallization process also offers the benefits of carrying out the energy-intensive emulsification step at a higher temperature and a lower viscosity, potentially reducing the energy consumption.

However, the stabilization mechanism can be system-specific, as the hydrophobicity and the chemistry of the solvent and the oil phase may change how MDG distributes in the emulsions, as evidenced by another study on MDG-stabilized emulsions [12]. In addition, other excipients and oil phase components commonly present can affect the crystallization of MDG. The resulting microstructures in such complex formulations are more difficult to study, as the presence of additional crystalline phases may obscure the observation of MDG crystals. Nevertheless, the fundamental understanding of the microstructure formation under different processing conditions and with different stabilizers obtained in this study is still highly valuable, as these insights provide the foundation for rational formulation and process design. 

## 5. Conclusions

This study investigated the effects of the processing conditions on various product attributes of emulsion-based semisolid formulations, with a model system consisting of mineral oil as the continuous oil phase, PG as the dispersed solvent phase and a stabilizer. Three stabilizers were investigated: MDG, which is widely used in the pharmaceutical industry; neat, distilled MG; and neat DG. The crystallization behavior of MDG in mineral oil was first studied to determine the crystallization temperatures of different components in MDG. MDG crystallized into two distinct crystal forms in the hydrophobic environment, which corresponded to the crystallization of MG and DG at different temperatures. Model polar liquid-in-oil emulsion systems were prepared with different emulsification temperatures determined based on the crystallization temperature of the individual component measured earlier, to cover both pre-crystallization and post-crystallization process regimes. The contribution of each component to stabilizing the emulsion samples was deciphered by studying emulsion samples containing either neat MG or DG as the only stabilizing agent. Product attributes, including rheology and physical stability, were characterized and correlated with the processing conditions. It was found that the post-crystallization-processed emulsions were more stable, with higher viscosity and more rigid structures compared to those in the emulsions prepared with pre-crystallization conditions for all stabilizing agents tested. This loss of stability can be partially salvaged by lowering the emulsification temperatures in the pre-crystallization process regime. In general, the MG-stabilized emulsions showed better physical stability and higher rheological properties than those of the DG-stabilized emulsions. This was attributed to the difference in crystal distribution in the emulsions, rather than polymorphism, as revealed using microscopy and XRD analysis. The XRD patterns of the different emulsion samples revealed that the crystallized glycerides in the final emulsion samples were all in the stable β polymorph. However, the microscopic images suggested that the crystal morphology and size can differ depending on the stabilizing agent used and the processing conditions. While MG adopted a plate-like morphology for all emulsification temperatures, DG formed spherulites when processed under pre-crystallization conditions and sheaves of plate-like crystals when processed with post-crystallization conditions. The crystal size also varied with emulsification temperature, where smaller crystals were formed at lower temperatures in the pre-crystallization regime, while post-crystallization processing also led to small crystal formation. Such differences in the emulsion microstructure can explain the observed difference in stability and rheological properties. For the MG-stabilized emulsions, self-hardening effects were observed over time, possibly due to restructuring and sintering of the crystal network, which strengthened the network further. 

## Figures and Tables

**Table 1:** Emulsion composition.

Ingredient	Function	Composition (%)
Propylene glycol (PG)	Aqueous/solvent phase	10
Mineral oil	Oil phase	80
MDG or MG or DG	Emulsifying agent	10 or 5

**Table 2:** Composition and emulsification temperature for all emulsion samples investigated in this study.

Emulsifying Agent	Emulsification Temperature (T_e_) (°C)	Composition		
		Mineral Oil	PG	Stabilizer
MDG	55	80	10	10
	45	80	10	10
	35	80	10	10
Distilled MG	60	80	10	5
	50	80	10	5
	40	80	10	5
	30	80	10	10
DG	45	80	10	10
	35	80	10	10

For the MDG-stabilized emulsions, a composition of mineral oil–PG–MDG at 80–10–5 was also used for microscopic analysis only (see Supporting Information). For the DG-stabilized emulsions, a composition of mineral oil–PG–DG at 80–10–5 was employed. However, no stable emulsions could be prepared under this composition.

**Table 3:** Instability indices and visual observation of emulsions stored in ambient conditions prepared under different processing conditions ^a^.

	MDG			MG				DG	
T_e_ (°C)	35	45	55	30	40	50	60	35	45
Instability Index (Fresh)	0.382 (0.003)	0.444 (0.006)	0.375 (0.002)	0.285 (0.002)	0.322 (0.006)	0.354 (0.010)	0.226 (0.003)	0.319 (0.006)	0.103 (0.008)
Instability Index (1 month)	0.400 (0.005)	0.431 (0.003)	0.360 (0.008)				0.129 (0.003)		
Visual Observation (1 month)	No PS ^b^	No PS	No PS	PS at day 20	PS at day 5	PS at day 1	No PS	PS at day 7	PS at day 14

^a^ Values in parentheses are standard deviation, n = 3. ^b^ PS = phase separation.

**Table 4:** Crossover stress for emulsion samples prepared under different processing conditions ^a^.

	MDG			MG				DG	
T_e_ (°C)	35	45	55	30	40	50	60	35	45
Crossover Stress (Fresh) (Pa)	11.49 (0.24)	2.79 (0.13)	13.78 (0.28)	1.45 (0.14)	0.92 (0.02)	0.58 (0.04)	1.11 (0.02)	1.10 (0.03)	3.08 (0.17)
Crossover Stress (1 month) (Pa)	14.29 (2.46)	3.53 (0.22)	16.89 (0.48)				16.85 (1.50)		

^a^ Values in parentheses are standard deviation (n = 3).

**Table 5:** Three-phase contact angles (*θ*) and representative images of sessile PG droplets against different solid substrates immersed in mineral oil ^a^.

	MDG	MG
θ (°)	146.3 (1.8)	124.6 (1.5)
Representative Image	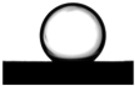	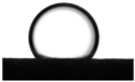

^a^ Values in parentheses are standard deviation (n = 3).

## Data Availability

The data that support the findings of this study are available from the corresponding author, Pui Shan Chow (ann_chow@isce2.a-star.edu.sg), upon reasonable request.

## Supplementary Materials

The following supporting information can be downloaded at https://www.mdpi.com/article/10.3390/pharmaceutics17091221/s1. Figure S1. DSC diffractogram of MDG dissolved in mineral oil at different compositions. Each measurement was triplicated, and the DSC diffractograms shown here are from a single run. Figure S2. DSC diffractogram of (a) distilled MG dissolved in mineral oil at 5 wt% and (b) diluted DG sample. Figure S3. Polarized microscopic images of emulsions containing mineral oil–PG–MDG at a composition of 85–10–5 with emulsification temperatures of (a) 35 °C, (b) 45 °C and (c) 55 °C after preparation. Red arrows indicate DG crystals as spherulitic DG crystals, and the yellow arrow indicates plate-like MG crystals. Figure S4. Viscosity curves for emulsion samples stabilized with (a) MDG (10 wt%), (b) MG (5 wt%) and (c) DG (10 wt%). Figure S5. Storage and loss moduli of the emulsion sample containing mineral oil–PG–MDG at a composition of 80–10–10 with an emulsification temperature of 55 °C. Figure S6. Microscopic images of emulsions containing mineral oil–PG–MDG at a composition of 80–10–10 with emulsification temperatures of (a) 35 °C, (b) 45 °C and (c) 55 °C and of emulsions containing mineral oil–PG–MG at a composition of 80–10–5 with an emulsification temperature of (d) 60 °C after 1 month of storage. Images in the left column were taken under a bright field, and images in the right column were taken under polarized light.

## Abbreviations

The following abbreviations are used in this manuscript:

MDG	Mono-and-diglyceride
MG	Monoglyceride
DG	Diglyceride
PG	Propylene glycol
API	Active pharmaceutical ingredient
PEG	Polyethylene glycol
DSC	Dynamic scanning calorimetry
XRD	X-ray diffraction
SAXS	Small-angle X-ray scattering
PLM	Polarized light microscopy
PS	Phase separation
MLCD	Medium- and long-chain diacylglycerol
