# Supplementary material for: Influence of Processing and Stabilizer Selection on Microstructure, Stability and Rheology of Emulsion-Based Semisolid Formulations
Source: Pharmaceutics. 2025 Sep 20;17(9):1221. doi: 10.3390/pharmaceutics17091221 (PMC12473458; doi:10.3390/pharmaceutics17091221)
Supplement: Supplementary file 1 [file pharmaceutics-17-01221-s001.zip › pharmaceutics-3848870-supplementary.pdf]

Supporting Information

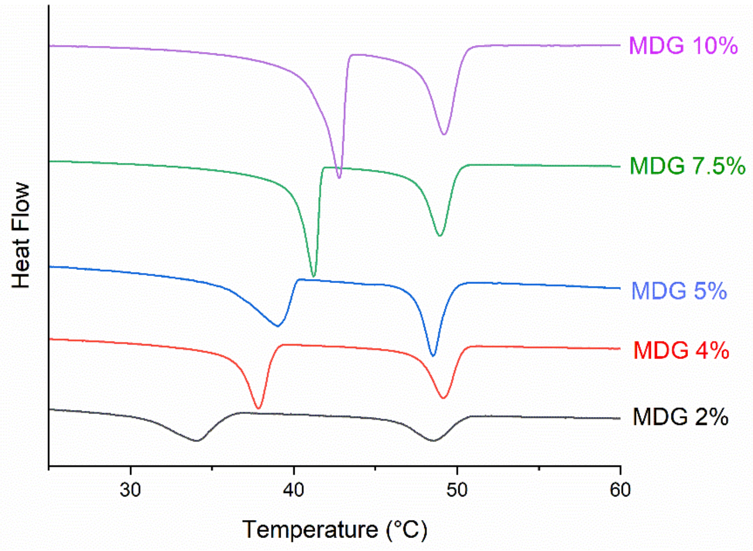

Figure S1. DSC Diffractogram of MDG dissolved in mineral oil at different compositions. Each measurement was triplicated and the DSC diffractograms shown here is from a single run.

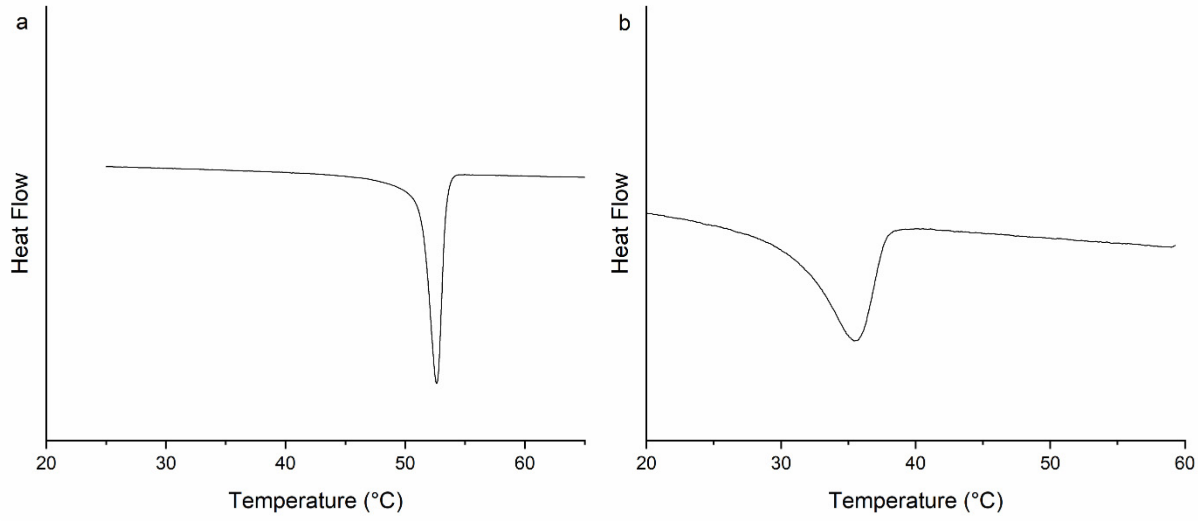

Figure S2. DSC Diffractogram of (a) Distilled MG dissolved in mineral oil at 5 wt% and (b) Diluted DG sample.

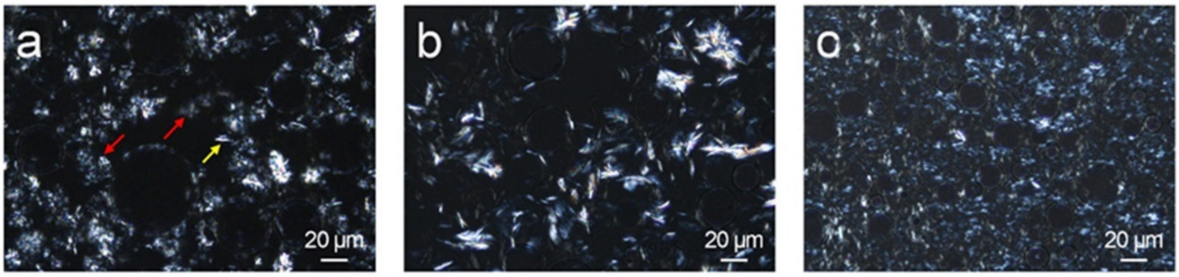

Figure S3. Polarized microscopic images of emulsions containing mineral oil – PG – MDG at a composition of 85 – 10 – 5 with emulsification temperatures of (a) 35 °C, (b) 45 °C and (c) 55 °C after preparation. Red arrows indicate spherulitic DG crystals and the yellow arrow indicates plate-like MG crystals. Scale bars are as shown.

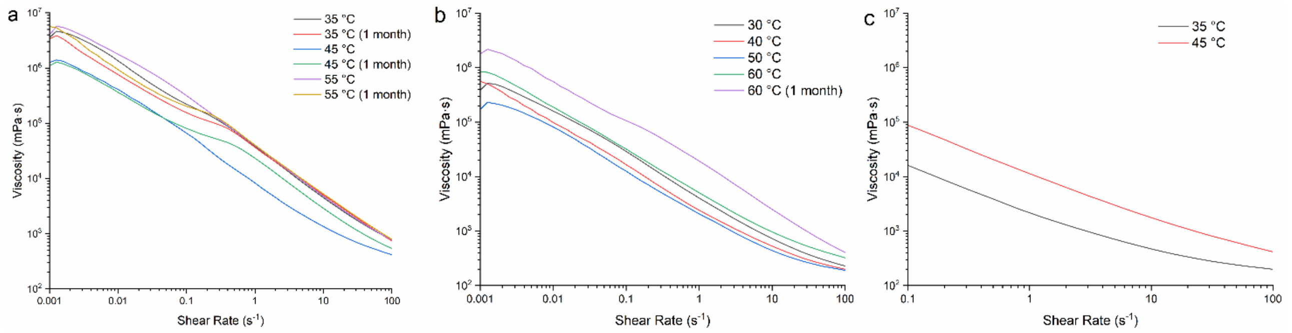

Figure S4. Viscosity Curve for emulsion samples stabilized with (a) MDG (10 wt%), (b) MG (5 wt%) and (c) DG (10 wt%).

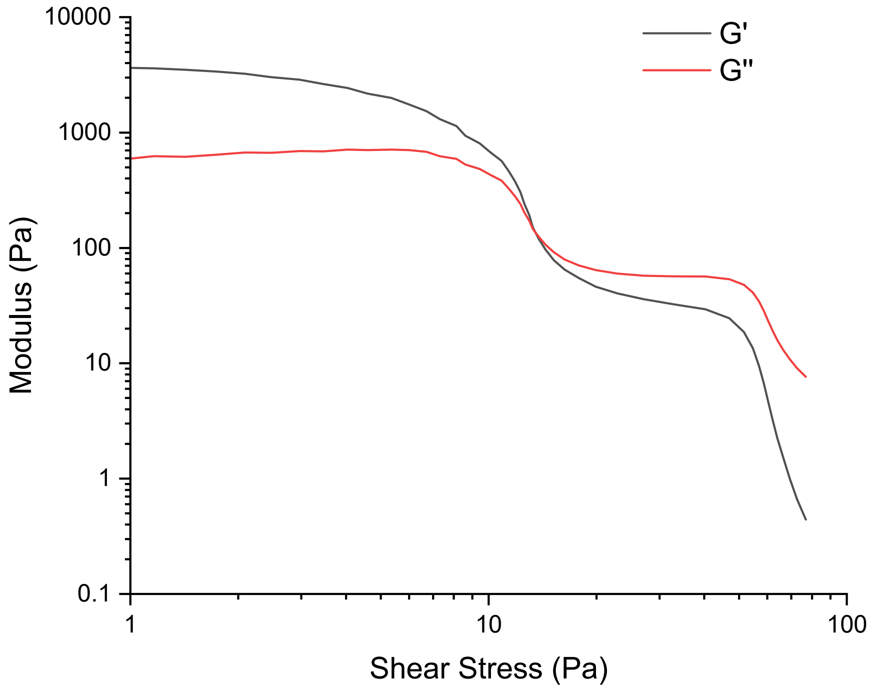

Figure S5. Storage and loss moduli of the emulsion sample containing mineral oil – PG – MDG at a composition of 80 – 10 – 10 with emulsification temperature of 55 °C.

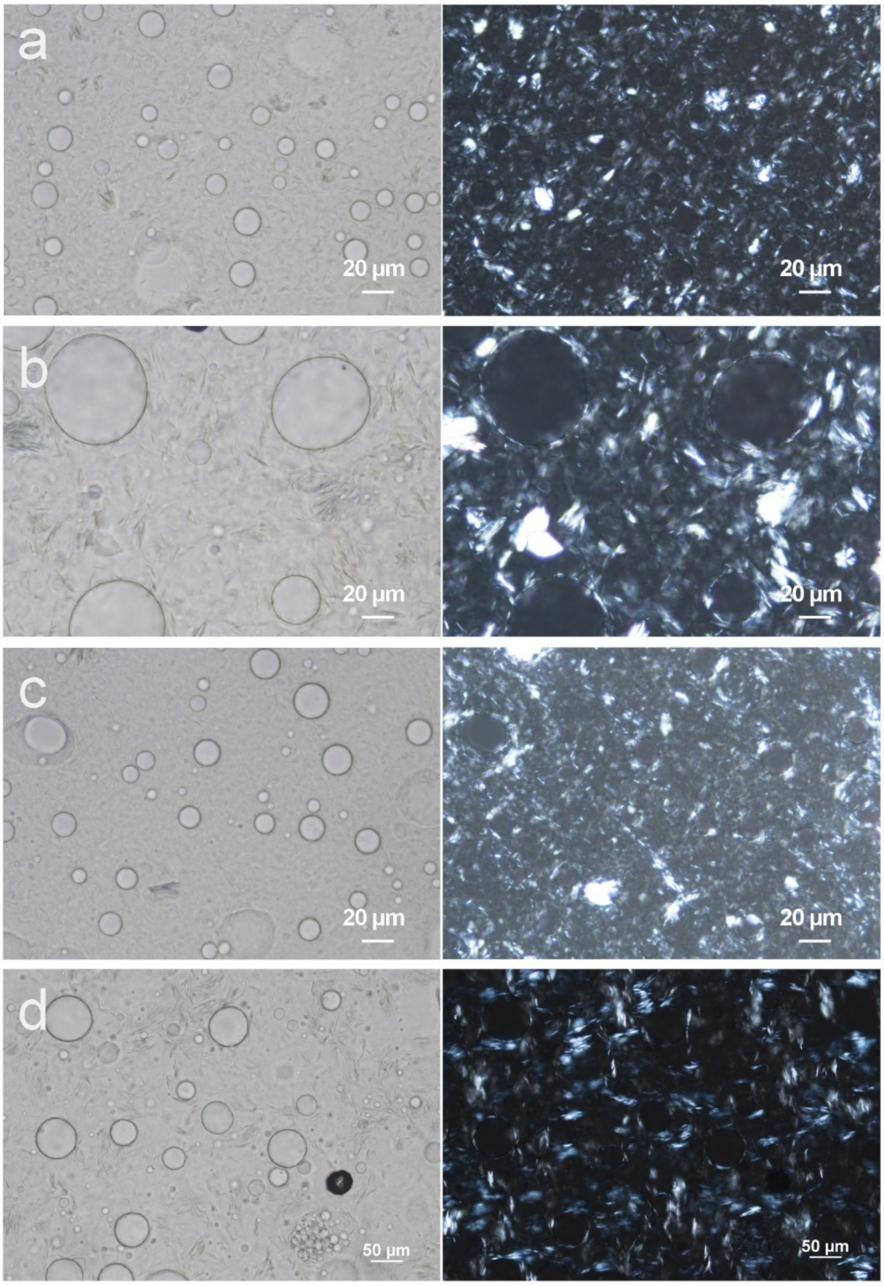

Figure S6. Microscopic images of emulsions containing mineral oil – PG – MDG at a composition of 80 – 10 – 10 with emulsification temperature of (a) 35 °C, (b) 45 °C and (c) 55 °C, and of emulsions containing mineral oil – PG – MG at a composition of 80 – 10 – 5 with emulsification temperature of (d) 60 °C after 1 month storage. Images in the left column were taken under bright field and images in the right column were taken under polarized light. Scale bars are as shown.
